# Fournier’s gangrene: prospective study of 34 patients in South Indian population and treatment strategies

**DOI:** 10.11604/pamj.2018.31.110.15495

**Published:** 2018-10-12

**Authors:** Vigneswara Srinivasan Sockkalingam, Elankumar Subburayan, Elango Velu, Sujith Tumkur Rajashekar, Anusha Mruthyunjaya Swamy

**Affiliations:** 1 Department of Surgery, Coimbatore Medical College, Coimbatore, India; 2 Department of Pharmacology, Narayana Medical College, Nellore, India; 3 Department of Medicine, Father Muller Medical College, Mangalore, India

**Keywords:** Fournier´s gangrene, necrotizing fasciitis, reconstructive procedure

## Abstract

**Introduction:**

Fournier's gangrene (FG) is a fulminant necrotizing fasciitis of the perineum and genitalia. The objective of this study was to study the etiology and microbiology associated with FG and to study the debridement and reconstructive procedures required in these patients.

**Methods:**

This was a prospective follow up study conducted from September 2011 to November 2012 at Coimbatore medical college hospital, Coimbatore, India. Patients presenting to the outpatient department and emergency department with the clinical diagnosis of FG were included in the study.

**Results:**

A total of 34 patients were studied in the study period. The mean age of presentation in years was 50±11.13. The male to female ratio was 33:1. The source of the infection was most commonly anorectal. Diabetes mellitus was the most common co morbid factor associated. Most commonly the disease was polymicrobial with escherichia coli being the commonest grown organism. The average number of wound debridement required was 2.9±1.42. Primary closure of the scrotal skin defect was the most common reconstructive procedure performed. Mortality associated with the disease in our series was 11.8%.

**Conclusion:**

Although FG is a relatively rare disease, it is still prevalent in Indian population. Incidence of FG in HIV patients is high, even though it is not the commonest of the co morbid condition. The mortality can be kept to minimal with aggressive medical and surgical management. Extensive raw area following the infection and wound debridement can be managed by simple reconstructive procedures with good outcome.

## Introduction

Fournier gangrene is a urologic emergency with a potentially high mortality rate. It is a rapidly progressing, polymicrobial necrotizing fasciitis of the perineal, perianal, and genital regions, with a mortality rate ranging from 15% to 50% [1, 2]. It is a relatively rare disease which is more common in poor socioeconomic strata of population. Most cases occur in 3^rd^ to 6^th^ decade of life. The disease occurs mainly in males. The overall incidence is approximately 1.6/100,000 males [2]. The disease is more prevalent among the diabetic and other immunosuppressed individuals. It is usually a polymicrobial infection caused by synergistic action of aerobic and anaerobic organisms [3, 4]. The microbes involved act synergistically via various enzymes like collagenase and hyaluronidase to invade and destroy tissue fascial planes. The disease process commonly has source of infection in the anorectum, urogenital tract, or skin of the genitalia. Mainstay of therapy involves early and aggressive surgical debridement in addition to adjuvant medical resuscitation along with appropriate antibiotics and treatment of existing co morbid conditions [5]. Reconstructive procedures are required in wider genital, perineal and abdominal wall defects. The goals of reconstructing Fournier defects are to provide protective coverage of the testes, preserve testicular function and gain acceptable cosmetic results with minimal associated morbidity and mortality. The literature regarding simple reconstructive procedures for the management of raw area following debridement of FG is limited. In this series we intend to study and analyze the etiology and microbiology of FG in the South Indian population. We also intend to study the feasibility of simple reconstructive procedures for the management of raw area due to the disease and debridement in the study population.

## Methods

This was a prospective study conducted from September 2011 to November 2012 at Coimbatore medical college hospital, Coimbatore, South India. Patients presenting to the outpatient department and emergency department with clinical diagnosis of FG were included in the study. Institution ethics committee clearance was obtained prior to the initiation of study. All patients were admitted and written informed consent was obtained before enrollment. International Conference on Harmonisation-Good Clinical Practice guidelines was followed. Patients presenting with necrotizing fasciitis of the genitalia and perineum were included and patients with only perianal/ischiorectal abscess were excluded from the study. The symptoms include scrotal pain, rapidly spreading cellulitis and erythema, fever and features of systemic toxicity. The diagnosis was made on basis of clinical scenario and not on histopathological results. After admission, stabilization and aggressive medical management was initiated. History of co-morbid conditions and history suggestive of primary source of infection was obtained. The extent of the disease was assessed clinically. Computed tomography (CT) of the abdomen and pelvis, ultra sonogram (USG) of the abdomen and pelvis and USG of the scrotum were done in select cases to define the extent of the disease and to identify the source of the infection. Blood investigations including complete blood count, assessment of electrolytes, blood urea nitrogen (BUN), creatinine, and blood glucose levels were done. Arterial blood gas (ABG) analysis was performed. Pus culture and sensitivity was done in all the patients. IV fluids were decided on individual case basis according to blood pressure, electrolytes and sugar levels. All patients were empirically started with third generation cephalosporins, aminoglycosides (Normal renal function patients) and metronidazole. Antibiotics were modified according to culture and sensitivity reports. Surgical wound debridement was done according to the extent of the disease. Reconstructive procedure was performed according to the raw area and available skin after the acute infection has subsided and after patient had become surgically fit. Debridements were done until most of the necrotic tissue and slough were removed. Few cases with larger affected area and increased risks of bleeding, debridement was done in a staged manner. Wound debridement was repeated if the wound was unhealthy and contained slough after initial debridement. Patients were followed up till discharge from the hospital and the results were reported. For data analysis descriptive statistics was used. Qualitative data are expressed as frequency and percentage. Quantitative data are expressed as mean and standard deviation.

## Results

**Figure 1:**
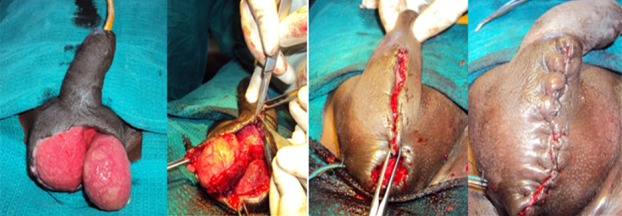
Primary closure of scrotal skin defect

**Figure 2:**
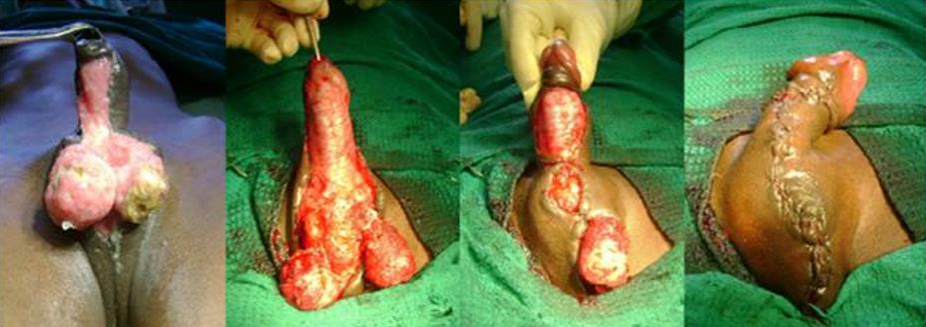
Primary closure + prepucial flap of penoscrotal skin defect

**Figure 3:**
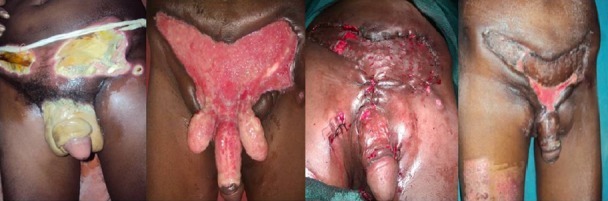
SSG + implantation of testis in thigh for extensive skin loss

Thirty four patients were treated in the Coimbatore medical college hospital during the specified study period. Highest numbers of patients were from fourth and fifth decade of life and the mean age of presentation was 50.03±11.13 years. The minimum age of the patient in our series was 32 years and the maximum age was 80 years. The mean age of presentation among deceased was 63±14.35 years and the mean age among survivors was 48.3±9.66 years. The male to female ratio was 33:1. There were no patients in the pediatric age group. The source of the disease was most commonly anorectal (35.3%) followed by genitourinary (20.6%) and dermatological (14.7%). The cause was idiopathic in 29.4% of cases (Table 1). Diabetes mellitus (38.2%) was the most common co morbid factor present followed by chronic alcoholism (20.6%), HIV (17.6%), chronic renal failure (8.9%), chicken pox (2.9%) and pulmonary tuberculosis (2.9%) (Table 2). The only female patient in our study had history of diabetes mellitus with recurrent cellulitis of groin. Most commonly the disease was polymicrobial (79.4%). The mean number of organisms present was 2.06±0.95. Three patients had maximum of 4 isolates per the culture and pus culture was sterile in 2 patients. Escherichia coli was isolated in 47.0% of patients followed by streptococci (41.1%) and klebsiella (35.3%). The bacteroides was the most commonly isolated anaerobe being isolated in 8.9% of patients (Table 3). In 18 out of 34 cases the disease was limited to the genitalia (52.9%). In addition to genitalia, perineum was also involved in 12 out of 34 (35.3%) patients. Genitalia, perineum and anterior abdominal wall were involved in 4 out of 34 (1.8%) patients. Multiple wound debridements were required in 29 patients. The average number of wound debridement needed per patient was 2.9±1.4. Single wound debridement was sufficient in 5 patients. Interestingly one patient required a maximum of 7 wound debridements. Reconstructive procedures for the raw area following wound debridement were required in 17 out of 34 patients (50%). 13 patients were managed by primary closure for scrotal skin defect (Figure 1). 2 patients needed local skin flap (prepucial skin flap) and primary closure for penoscrotal skin defect (Figure 2). One patient needed split skin grafting for abdominal wall skin defect in addition to primary closure for scrotal skin defect. One another patient required split skin grafting for penile and abdominal wall skin defect + implantation of bilateral testis in the thigh (Figure 3). Mortality was associated with 4 patients in our series (11.8%).

**Table 1:** Source of infection

Source of infection	Number of patients (n)	Percentage of patients (%)
Anorectal	12	35.3
Urogenital	7	20.6
Dermatological	5	14.7
Idiopathic	10	29.4

**Table 2:** Comorbid conditions

Comorbid conditions	Number of patients (n)	Percentage of patients (%)
Diabetes Melitus	13	38.2
Chronic Alcholism	7	20.6
HIV	6	17.6
Chronic Renal Failure	3	8.9
Tuberculosis	1	2.9
Chickenpox	1	2.9
Nil	3	8.9

**Table 3:** Composition of the isolated organisms

Isolated microbe	Number of patients (n)	Percentage of patients (%)
Escherichia Coli	16	47.0
Streptococcus	14	41.1
Klebsiella	12	35.3
Pseudamonas	9	26.5
Staphylococcus	6	17.7
Enterococcus	4	11.8
Proteus	4	11.8
Bacteroides	3	8.9
Acinetobacter	2	5.9

NOTE: Since most of the cases are polymicrobial (79.4%) the the percentage of cases for each microbes when added together will be more than 100.

## Discussion

The history of FG goes back to 18th century when baurienne originally described an idiopathic, rapidly progressive soft-tissue necrotizing process that resulted in gangrene of the male genitalia and perineum [6]. However, the disease was named after Jean-Alfred Fournier, a French venereologist who described the disease in detail. In 1883 he presented a series of five cases of perineal and scrotal gangrene in otherwise healthy males [7]. It is a relatively uncommon disease. The real incidence of the disease is unknown. In a large population based study FG represented less than 0.02% of hospital admissions. Poor socioeconomic status contributes to the development of FG. Most cases occur in the age group of 30-60 years. The mean age of the patients in our series was 50.0±11.13 years. In concordance with the available literature [8, 9] mean age among survivors was 48.3±9.66 years, which is significantly lower than mean age among deceased which was 63±14.35 years. Although pediatric population can be affected [10] there were no pediatric cases in our series. Male to female ratio in a large series by Eke et al is approximately 10:1 [2]. In our series there was only one female patient out of 34. The lower incidence of the disease in females can be explained by better drainage of perineal secretions. Although originally described as idiopathic, FG do have an identifiable cause in most of the cases (75-95%).The disease process commonly has source of infection from the anorectum, the urogenital tract, or the skin of the genitalia. Localized infection nearer to a portal of entry is often the inciting event in the development and progression of FG. Anorectal origin was the most common source of infection in many previous studies [11]. Likewise in our series also anorectal focus was the most common source of infection.

As is the case with most of the published studies [3, 12-14], diabetes mellitus was the most important coexisting factor in our study(38.2%). In one study by Smith et al, chronic alcoholism was found to be the most common co morbid condition [15]. In a few studies from Africa HIV is the most important co morbid condition [16, 17]. In our series also we had significant proportion of HIV infected patients (17.6%) even though it was not the most common cause. Hejase et al & Ferreira et al had polymicrobial isolation in 90% and 82.9% of cases respectively [1, 2]. In our present series polymicrobial infection was found in 79.4% of cases. The mean microbial number identified in culture tests were around 2 to 3 in various series [12, 18]. In our present series the average number of isolates found per case was 2.06±0.95. Escherichia coli and streptococcus were the most commonly isolated aerobe. In a study of 43 patients by Ferreira et al, single debridement was sufficient in 35 patients, 7 patients were debrided twice and 1 patient was debrided thrice [2]. But contrastingly according to Chawla et al the average number of debridement required was 3.5 procedures per patient [19]. In our series also the average numbers of debridement needed were 2.9±1.42 /patient. Delayed presentation to the hospital with extensive disease involvement in our study population could be the reason for need of multiple debridements. In a few series 100% mortality has been reported when surgical debridement was not performed [20-22].

The percentage of patients requiring colostomy in many series is around 15% [3]. A few series have reported that performing a diversion colostomy is associated with an increased mortality [23]. None of the patients in our series had to undergo either diversion colostomy or orchiectomy. After the acute phase of the infection has subsided the scrotum can be left alone in many patients for healing by secondary intention as it has remarkable capacity to regenerate [24]. The reconstructive procedures if indicated can be performed in the same admission [25] or after the resolution of acute infectious process. In our series all the reconstructive procedures were performed after the resolution of acute infectious process in 2 to 4 weeks time. In a series by Prakash et al, in most of the cases cover was provided with scrotal skin for the scrotal defects and the penile skin defects were covered with inner layer of prepuce [26]. Our experiences also go hand in hand with theirs. In our series simple reconstructive procedures like primary closure of the scrotal raw area and primary closure combined with prepucial skin cover for penoscrotal raw area was successfully done in 13 (35.8%) and 2 (5.9%) patients respectively. More elaborate reconstructive procedures were required only in 2 (5.9%) patients. In 17 patients (50 %) no reconstructive procedures were required. Even though skin loss appeared extensive in a few patients these simple reconstructive procedures yielded a good outcome. The mortality rate is lower than that of others forms of necrotizing fasciitis. It is probably because, the scrotal area allows for a relatively more efficient surgical debridement [2]. The mortality rate of our series was 11.8%. The mortality rate was on par with most of the studies conducted internationally. The limitation in our study is small sample size and lack of long term follow up.

## Conclusion

Although FG is a rare disease but still prevalent in Indian population. In our patients, the source of the disease was most commonly anorectal and diabetes mellitus was the most important comorbid condition. Escherichia coli and streptococcus were the most commonly isolated aerobe. The incidence of FG in HIV patients is significant, even though it is not the commonest of the co morbid condition. The mortality can be kept to minimal with aggressive medical and surgical management. Extensive raw area following the infection and wound debridement can be managed by simple reconstructive procedures with good outcome. Complex reconstructive surgical procedures are rarely required.

### What is known about this topic

- FG is the fulminant necrotizing fasciitis of the perineum and genitalia with high mortality;
- Without appropriate treatment the disease carries high mortality.

### What this study adds

- Data from south Indian population;
- Simple reconstructive procedures can be performed in most of the cases with good outcome;
- HIV is a significant co morbid factor even though it is not the most common one in the South Indian population.

## Competing interests

All authors declare no competing interest.
